# Ambulatory blood pressure as risk factor for long-term kidney function decline in the general population: a distributional regression approach

**DOI:** 10.1038/s41598-023-41181-7

**Published:** 2023-08-31

**Authors:** Bjørn O. Eriksen, Matteo Fasiolo, Ulla D. Mathisen, Trond G. Jenssen, Vidar T. N. Stefansson, Toralf Melsom

**Affiliations:** 1 Metabolic and Renal Research Group, UiT The Arctic University of Norway, Tromsø, Norway; 2 Section of Nephrology, Clinic of Internal Medicine, University Hospital of North Norway, Tromsø, Norway; 3 School of Mathematics, University of Bristol, Bristol, UK; 4 Department of Transplant Medicine, Oslo University Hospital and University of Oslo, Oslo, Norway

**Keywords:** Physiology, Diseases, Health care, Medical research, Nephrology, Pathogenesis, Risk factors

## Abstract

The results of randomized controlled trials are unclear about the long-term effect of blood pressure (BP) on kidney function assessed as the glomerular filtration rate (GFR) in persons without chronic kidney disease or diabetes. The limited duration of follow-up and use of imprecise methods for assessing BP and GFR are important reasons why this issue has not been settled. Since a long-term randomized trial is unlikely, we investigated the association between 24-h ambulatory BP (ABP) and measured GFR in a cohort study with a median follow-up of 11 years. The Renal Iohexol Clearance Survey (RENIS) cohort is a representative sample of persons aged 50 to 62 years without baseline cardiovascular disease, diabetes, or kidney disease from the general population of Tromsø in northern Norway. ABP was measured at baseline, and iohexol clearance at baseline and twice during follow-up. The study population comprised 1589 persons with 4127 GFR measurements. Baseline ABP or office BP components were not associated with the GFR change rate in multivariable adjusted conventional regression models. In generalized additive models for location, scale, and shape (GAMLSS), higher daytime systolic, diastolic, and mean arterial ABP were associated with a slight shift of the central part of the GFR distribution toward lower GFR and with higher probability of GFR < 60 mL/min/1.73 m^2^ during follow-up (p < 0.05). The use of a distributional regression method and precise methods for measuring exposure and outcome were necessary to detect an unfavorable association between BP and GFR in this study of the general population.

## Introduction

High blood pressure (BP) is the leading risk factor for death and loss of disability-adjusted life years globally and is an important risk factor for end-stage kidney disease (ESKD)^1^. Whereas randomized controlled trials (RCTs) have established hypertension as a cause of cardiovascular disease beyond a reasonable doubt, similar high-quality evidence does not exist for the prevention of chronic kidney disease (CKD) by treating primary hypertension in persons without diabetes^2–6^. Indeed, at least two RCTs have found an adverse effect of intensified antihypertensive treatment on the glomerular filtration rate (GFR)^4,5^. Although this may have been caused by short-term hemodynamic changes that may ultimately lead to beneficial long-term effects, this remains unproven because of the limited duration of follow-up in the RCTs^2–5^.

The lack of definitive evidence for the causal association between high BP and loss of kidney function has raised doubts about whether nonmalignant primary hypertension is a cause of CKD in persons without diabetes. In a study of kidney biopsies from live kidney donors by Denic et al., mild hypertension was not associated with the number of nephrons, the single-nephron glomerular filtration rate (GFR), or the total GFR^7^. In the longitudinal population-based Renal Iohexol Clearance Survey (RENIS), we did not find an association between elevated BP and accelerated mean GFR decline in the general middle-aged population over a median follow-up of 5.6 years^8,9^. We hypothesized that additional genetic and environmental factors are necessary for elevated BP to cause CKD in some individuals after an even longer observation period.

In the present study, we investigated this hypothesis by analyzing baseline 24-h ambulatory blood pressure (ABP) as a risk factor for change in the GFR measured as iohexol clearance after a follow-up of more than ten years. Since conventional least squares regression methods only analyze changes in the mean of the GFR distribution while assuming its other properties to be constant, we used distributional regression to analyze the associations between ABP and the time change of different percentiles of the GFR distribution^10^.

## Methods

### Study population

**Figure 1:**
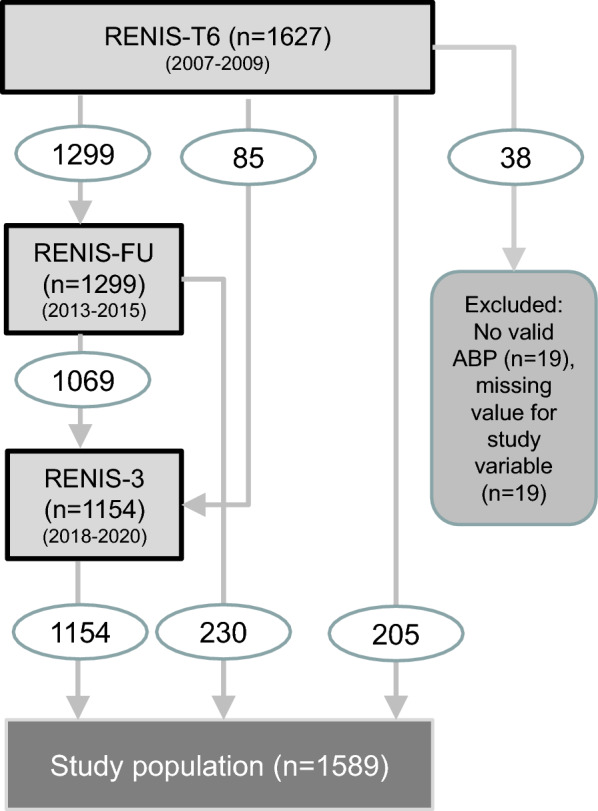
Persons from the Renal Iohexol Clearance Survey (RENIS) cohort were included in the present investigation. The numbers in ovals represent the numbers of persons from one wave of the investigation included in the next. RENIS-T6 the baseline investigation; RENIS-FU, the first follow-up; RENIS-3, the last follow-up; ABP, ambulatory blood pressure.

The Renal Iohexol Clearance Survey (RENIS) is a substudy of the Tromsø Study. The Tromsø Study has invited random samples of the general population of the municipality of Tromsø in northern Norway to a series of repeated health surveys^11^. The RENIS cohort was recruited from all persons between 50 and 62 years of age examined in the sixth Tromsø Study. All persons without self-reported cardiovascular disease, kidney disease or diabetes mellitus were invited, and 1627 persons were included in random order until a prespecified target was met. The cohort underwent measurements of plasma iohexol clearance at baseline in 2007–2009 (RENIS-T6), in 2013–2015 (RENIS-FU) and in 2018–2020 (RENIS-3) (Fig. 1). The inclusion process has been described in detail previously^12^. All included persons were invited to ABP measurement at baseline, and everybody with a valid measurement was eligible for the present study (Fig. 1). The GFR measurements of a small random sample who had an extra GFR measurement for the purpose of assessing day-to-day variation in RENIS-FU were also included in the analyses.

This study complied with the Declaration of Helsinki and was approved by the Regional Committee for Medical and Health Research Ethics of Northern Norway. All subjects provided a written informed consent.

### Data

The investigations were performed at the Clinical Research Unit of the University Hospital of North Norway. The participants answered questionnaires that included questions about previous diseases, alcohol use, smoking habits, and current medication. Alcohol use was analyzed as a dichotomous variable for the weekly use of alcohol or not. Smoking was analyzed as the number of cigarettes per day currently used. Antihypertensive medication was analyzed as separate dichotomous variables for the use of ACE inhibitors, A2 receptor blockers, beta-blockers, calcium blockers, diuretics or other antihypertensives.

### Measurements

#### Iohexol clearance

GFR was measured as single-sample plasma iohexol clearance, which has been validated against gold standard methods^13,14^ and has been described in detail previously^12,15^. Five millilitres of iohexol were injected intravenously and a sample obtained for iohexol measurement at the optimal sampling time for each person calculated by Jacobsson’s equation^16^. GFR was calculated by a numerical solution of Jacobsson’s three equation ^16^. To avoid confounding from changes in body size, absolute GFR in mL/min was used. Body surface area-indexed GFR was analyzed in a sensitivity analysis^17^. Body surface area was estimated by the equation of DuBois and DuBois^17^.

#### Blood pressure measurements

Twenty-four-hour ABP was initiated at the day of the baseline GFR measurement using the Spacelab 90207 (Spacelab Inc., Redmond, Washington, USA) as described previously^18^. The criteria for a valid ABP measurement were adopted from the International Database on Ambulatory Blood Pressure in Relation to Cardiovascular Outcome study^19^. Attended office BP was measured after 2 min of rest in the seated position with an automated device (model UA 799; A&D, Tokyo, Japan) by a study nurse^18^. The daytime to nighttime systolic and diastolic dips were analyzed as one minus the ratio of the mean nighttime to daytime systolic BP (SBP) or diastolic BP (DBP). Ambulatory mean arterial pressure (MAP) was defined as DBP + ((SBP − DBP)/3).

Office hypertension was defined as office SBP ≥ 140 mmHg or office DBP ≥ 90 mmHg or the use of antihypertensive medication according to the guidelines of the European Society of Hypertension^20^.

#### Other baseline measurements

Fasting serum glucose, creatinine, cystatin C, triglycerides, and LDL- and HDL-cholesterol were measured with standard methods as described previously^18^. The urine albumin-creatinine ratio (ACR) was measured as the median of ACR measured on three separate days^21^. Serum creatinine was measured using an enzymatic assay standardized to the isotope dilution mass spectrometry method (CREA Plus, Roche Diagnostics, GmbH, Mannheim, Germany). Cystatin C was measured by a particle-enhanced turbidimetric immunoassay (Gentian, Moss, Norway) calibrated to the international reference ERM-DA471/IFCC as described previously^22^. Estimated GFR (eGFR) was calculated using the original Chronic Kidney Disease Epidemiology Collaboration equations published in 2009 and 2012 (eGFR_crea_, eGFR_cys_ and eGFR_cyscrea_)^23,24^.

### Statistical methods

The baseline characteristics of the cohort are given as the mean (standard deviation) or median (interquartile range) for ABP < or ≥ 130/80, the threshold for hypertensive 24-h ABP according to the European Society of Hypertension^20^. Differences across the ABP levels were analyzed with two-sample t-tests, Wilcoxon rank-sum tests or tests of proportion as appropriate.

We first investigated the relationship between mean GFR and the BP components with general additive mixed models (GAMM)^25,26^. GAMMs are a generalization of linear mixed models where nonlinear effects of the independent variables can be modeled. The reason for using GAMMs and not linear mixed models was that a previous investigation in the RENIS cohort found sex-specific nonlinear relationships between mean GFR and time^12^. Accordingly, we adjusted for these relationships in the GAMMs.

The GAMMs had GFR as the dependent variable and a linear term for each BP component as the independent variable in separate models. The models included a random intercept and slope and an unstructured covariance matrix. We analyzed office and ambulatory daytime and nighttime SBP, DBP, and MAP as well as the systolic and diastolic nighttime BP dips. We included both the linear main effects of these BP components and their interaction with time. The coefficient for this interaction represented the association between the component and the GFR change rate. A negative sign for the coefficient signified a steeper GFR decline. The time variable was defined as years since baseline. In addition to the sex-specific nonlinear time variables, we included two sets of linear baseline adjustment variables, including their interactions with time: Model 1: sex and sex-specific variables for baseline age, body weight, height, and dichotomous variables for each class of antihypertensive medication. Model 2: As Model 1 with the addition of pulse frequency, fasting glucose, triglycerides, LDL- and HDL-cholesterol, number of cigarettes currently smoked per day, and a dichotomous variable for weekly alcohol use. Model 3: As Model 2 with the addition of ACR. All study participants were included in the GAMM analyses regardless of whether they were examined at follow-up because mixed models allow for missing observations at one or more points in time^27,28^.

Next, we analyzed the associations between ABP and the time change of the GFR distribution in generalized additive models for location, scale, and shape (GAMLSS). Conventional least squares regression methods analyze the effect of exposures on the mean of outcomes, whereas other aspects of their probability distributions are assumed to be independent of the exposures. GAMLSS relaxes these assumptions and analyzes the effects of the exposure on the total outcome distribution^25,26,29^. The GFR distribution used in this investigation was the sinh-arcsinh (SHASH) distribution^30^, which is specified by the four parameters location, scale, skewness, and tailweight. Variations of these four parameters permit greater flexibility in the probability distribution that can be modeled than the usual normal distribution (see Online Resource and Fig. S1). The GAMLSS models each of the four parameters as a nonlinear function of the ABP component and of their interaction with time as independent variables. Accordingly, the main difference between GAMLSS and conventional regression methods is that GAMLSS analyzes four dependent variables (location, scale, skewness, and tailweight) simultaneously in one model, whereas other methods only analyze one dependent variable (the mean). The location, scale, and tailweight parameters are related (but not exactly equivalent) to the mean, standard deviation, and kurtosis (see Online Resource and Fig. S1). In the function for the location, we included a random intercept and adjustments as in Model 2 in the GAMM above, except that we used one dichotomous variable for the use of any antihypertensive medication to simplify the model. For the same reason, we restricted adjustments in the functions for scale, skewness, and tailweight to sex-specific nonlinear functions of the time variable. We investigated day- and nighttime SBP, DBP, and MAP in separate GAMLSS.

To find the simplest model consistent with the data, we compared the fit of models with the four SHASH parameters specified above with models where one or more of the nonlinear functions for scale, skewness or tailweight were replaced by a constant. Models with all eight possible combinations of replacement by a constant for these three parameters were examined. The Akaike Information Criterion (AIC) was used to compare the fit of the models^31^. The p-value for the nonlinear interaction between the ABP component and time for each SHASH parameter in each best-fitting model was used to judge whether there was a time-dependent association between the ABP component and the development of the GFR distribution. The finding of an association implied that the corresponding ABP component was a risk factor for time change in the GFR distribution. The p-value for the nonlinear main effect of an ABP component in each best-fitting model was used to judge whether there was a time-independent cross-sectional association between the BP component and GFR.

STATA/MP 17.0 (www.stata.com) and R version 3.6.3 (www.r-project.org) were used for the analyses in this study. The mgcv and mgcViz packages of R were used for the analyses with GAMM and GAMLSS^25,26^. Statistical significance was set at p < 0.05.

## Results

Valid ABP measurements were obtained for 1608 (99%) of the 1627 persons included at baseline. Because of missing values for some of the adjustment variables for 38 persons (Table S1), the study population consisted of 1589 (98%) complete cases (Table S1, Fig. 1). Of these 1589 persons, 1299 had repeated GFR measurements in RENIS-FU and 1154 in RENIS-3. In addition, a random sample of 85 of the participants in RENIS-FU had an extra measurement to measure the day-to-day variation in GFR. Accordingly, the total number of GFR measurements was 4127. Reasons for not attending and comparisons of investigated persons with all eligible persons have been published previously^12^. The median (IQR) (range) follow-up was 10.7 (6.4–11.3) (0–12.8) years.

Most baseline characteristics (Table 1), including mGFR, differed between the two categories of ABP (p < 0.05), but not eGFR_crea_, eGFR_cys_, eGFR_cyscrea_, smoking, alcohol use or LDL-cholesterol.

**Table 1:** Baseline characteristics of the RENIS cohort according to baseline ambulatory blood pressure.

Baseline characteristic	24 h ambulatory blood pressure						
	All (n = 1589)		≥ 130/80 (n = 604)		< 130/80 (n = 985)		P-value
Age, years	58.1	(3.8)	58.3	(3.8)	57.9	(3.9)	0.10
Male gender, %	779	(49%)	409	(68%)	370	(38%)	< 0.001
Body weight, kg	79.7	(14.4)	84.0	(14.3)	77.0	(13.9)	< 0.001
Height, cm	170.6	(8.7)	172.9	(8.5)	169.2	(8.6)	< 0.001
Body surface area (m^2^)	1.91	(0.20)	1.98	(0.19)	1.87	(0.19)	< 0.001
Body mass index, kg/m^2^	27.3	(4.0)	28.0	(3.8)	26.8	(4.1)	< 0.001
Body mass index ≥ 30 kg/m^2^, n(%)	360	(23%)	161	(27%)	201	(20%)	0.004
Current smoker, n (%)	337	(21%)	120	(20%)	217	(22%)	0.31
Use of alcohol at least weekly, n(%)	433	(27%)	179	(30%)	254	(26%)	0.09
LDL cholesterol, mmol/L	3.7	(3.1)	3.7	(3.2)	3.6	(3.0)	0.06
HDL cholesteroll, mmol/L	1.5	(1.2)	1.4	(1.1)	1.5	(1.3)	< 0.001
Fasting triglycerides, mmol/L	1.00	(0.80 to 1.50)	1.20	(0.80 to 1.60)	1.00	(0.70 to 1.30)	< 0.001
Fasting glucose, mmol/L	5.30	(5.00 to 5.60)	5.40	(5.10 to 5.70)	5.20	(4.90 to 5.60)	< 0.001
Ambulatory blood pressure, mmHg							
24 h systolic	123.3	(12.3)	134.7	(9.7)	116.4	(7.7)	< 0.001
24 h diastolic	76.5	(8.1)	84.0	(6.1)	71.8	(5.2)	< 0.001
24 h mean arterial	92.1	(9.0)	100.9	(6.3)	86.7	(5.4)	< 0.001
Daytime systolic	130.2	(13.2)	141.9	(10.6)	123.0	(8.7)	< 0.001
Daytime diastolic	82.1	(8.7)	89.7	(6.8)	77.4	(6.0)	< 0.001
Daytime mean arterial	98.1	(9.6)	107.1	(7.0)	92.6	(6.3)	< 0.001
Nighttime systolic	111.0	(12.4)	121.2	(11.1)	104.8	(8.5)	< 0.001
Nighttime diastolic	66.4	(8.5)	73.3	(7.3)	62.2	(5.9)	< 0.001
Nighttime mean arterial	81.3	(9.3)	89.3	(7.8)	76.4	(6.2)	< 0.001
Office blood pressure, mmHg							
Systolic	129.6	(17.7)	142.2	(15.9)	121.9	(13.9)	< 0.001
Diastolic	83.4	(9.8)	90.2	(8.5)	79.2	(8.1)	< 0.001
Mean arterial	98.8	(11.8)	107.5	(9.9)	93.4	(9.4)	< 0.001
Office hypertension, n(%)	676	(43%)	439	(73%)	237	(24%)	< 0.001
Antihypertensive medication, n(%)	295	(19%)	158	(26%)	137	(14%)	< 0.001
ACE inhibitor, n(%)	29	(2%)	20	(3%)	9	(1%)	0.001
A2 blocker, n(%)	136	(9%)	69	(11%)	67	(7%)	0.001
Betablocker, n(%)	72	(5%)	30	(5%)	42	(4%)	0.51
Diuretic, n(%)	146	(9%)	76	(13%)	70	(7%)	< 0.001
Calcium blocker, n(%)	81	(5%)	47	(8%)	34	(3%)	< 0.001
Other antihypertenives, n(%)	2	(0%)	1	(0%)	1	(0%)	0.73
Urinary albumin-creatinine ratio							
0–29, mg/g	1566	(99%)	592	(98%)	974	(99%)	0.18
30–299, mg/g	22	(1%)	11	(2%)	11	(1%)	
≥ 300 mg/g	1	(0%)	1	(0%)	0	(0%)	
Absolute measured GFR, mL/min	104.0	(20.1)	109.2	(20.8)	100.8	(18.9)	< 0.001
Measured GFR, mL/min/1.73 m^2^	93.9	(14.4)	95.5	(14.7)	93.0	(14.2)	0.001
Creatinine-based estimated GFR^a^, mL/min/1.73 m^2^	94.9	(9.5)	94.6	(9.5)	95.0	(9.5)	0.41
Cystatin C-based estimated GFR^a^, mL/min/1.73 m^2^	105.4	(12.4)	105.5	(12.4)	105.4	(12.4)	0.85
Creatinine and cystatin C-based estimated GFR^a^, mL/min/1.73 m^2^	103.0	(11.4)	102.7	(11.3)	103.2	(11.5)	0.37

*RENIS* the Renal Iohexol-clearance Survey. Estimates are given as mean (standard deviation), median (interquartile range) or n(percent).

^a^GFR estimated with equations published by the Chronic Kidney Disease Epidemiology Collaboration (reference 21).

### Associations of blood pressure components with the mean GFR change rate

There were no statistically significant linear associations of any of the investigated BP components with the mean GFR change rate in the GAMMs in the fully adjusted model (Table 2). Sensitivity analyses after excluding observations with self-reported incident CVD during follow-up, after excluding persons with antihypertensive treatment and with body surface area adjusted GFR gave similar results (Supplementary Results, Tables S2 and S3).

**Table 2:** Linear associations between the mean GFR change rates and baseline blood pressure components in generalized additive mixed models. The RENIS cohort.

Blood pressure component	Model 1					Model 2					Model 3				
	Beta coefficient, mL/min/year (95% CI)				P-value	Beta coefficient, mL/min/year (95% CI)				P-value	Beta coefficient, mL/min/year (95% CI)				P-value
Ambulatory blood pressure, per 10 mmHg increase															
24 h															
Systolic	− 0.07	− 0.13	to	− 0.01	0.02	− 0.05	− 0.11	to	0.01	0.09	− 0.04	− 0.10	to	0.01	0.14
Diastolic	− 0.05	− 0.14	to	0.03	0.23	− 0.04	− 0.13	to	0.05	0.39	− 0.03	− 0.12	to	0.06	0.49
Mean arterial pressure	− 0.07	− 0.15	to	0.01	0.08	− 0.05	− 0.13	to	0.03	0.19	− 0.05	− 0.13	to	0.04	0.27
Daytime															
Systolic	− 0.05	− 0.10	to	0.01	0.10	− 0.03	− 0.08	to	0.02	0.27	− 0.03	− 0.08	to	0.03	0.36
Diastolic	− 0.02	− 0.10	to	0.06	0.64	− 0.01	− 0.09	to	0.08	0.86	0.00	− 0.09	to	0.08	0.99
Mean arterial pressure	− 0.04	− 0.11	to	0.03	0.30	− 0.02	− 0.10	to	0.05	0.53	− 0.02	− 0.09	to	0.06	0.67
Nighttime															
Systolic	− 0.07	− 0.13	to	− 0.02	0.01	− 0.06	− 0.11	to	0.00	0.05	− 0.05	− 0.11	to	0.01	0.09
Diastolic	− 0.08	− 0.16	to	0.01	0.07	− 0.07	− 0.15	to	0.01	0.10	− 0.06	− 0.15	to	0.02	0.15
Mean arterial pressure	− 0.09	− 0.16	to	− 0.01	0.02	− 0.07	− 0.15	to	0.00	0.07	− 0.06	− 0.14	to	0.01	0.10
Daytime to nighttime blood pressure dip^a^, per 0.10 increase															
Systolic	− 0.08	− 0.19	to	0.03	0.15	− 0.07	− 0.17	to	0.04	0.21	− 0.06	− 0.17	to	0.04	0.23
Diastolic	− 0.09	− 0.18	to	0.01	0.07	− 0.08	− 0.17	to	0.01	0.10	− 0.07	− 0.17	to	0.02	0.11
Office blood pressure, per 10 mmHg increase															
Systolic	− 0.04	− 0.08	to	0.00	0.03	− 0.04	− 0.08	to	0.01	0.010	− 0.03	− 0.07	to	0.01	0.14
Diastolic	− 0.05	− 0.12	to	0.03	0.23	− 0.04	− 0.12	to	0.03	0.26	− 0.04	− 0.12	to	0.04	0.32
Mean arterial pressure	− 0.06	− 0.12	to	0.01	0.08	− 0.05	− 0.11	to	0.02	0.14	− 0.04	− 0.11	to	0.02	0.19

Each line for each model in the table represents the linear association between the blood pressure component in the first column and the GFR change in a separate generalized additive mixed model. Model 1 was adjusted for sex and sex-specific variables for baseline age, body weight, height, dichotomous variables for each class of antihypertensive medication and sex-specific non-linear terms for the time variable. Model 2: As model 1 and in addition pulse frequency, fasting glucose, triglycerides, LDL- and HDL-cholesterol, number of cigarettes currently smoked per day, and a dichotomous variable for the weekly alcohol use. Model 3: As model 2 and in addition the urinary albumin-creatinine ratio.

*RENIS* the Renal Iohexol-clearance Survey, *GFR* glomerular filtration rate, *CI* confidence interval.

*Calculated as one minus the ratio of the mean nighttime to daytime systolic or diastolic blood pressure.

### Nonlinear associations of ambulatory blood pressure with the time change in the GFR distribution

GAMLSS with nonlinear functions for location, scale, and skewness, but with a constant tailweight parameter, had the lowest AIC and best fit for all of the ABP components except nighttime DBP (Model G, Table S4). For nighttime DBP, a model with both constant skewness and tailweight had the lowest AIC (Model C, Table S4).

**Figure 2:**
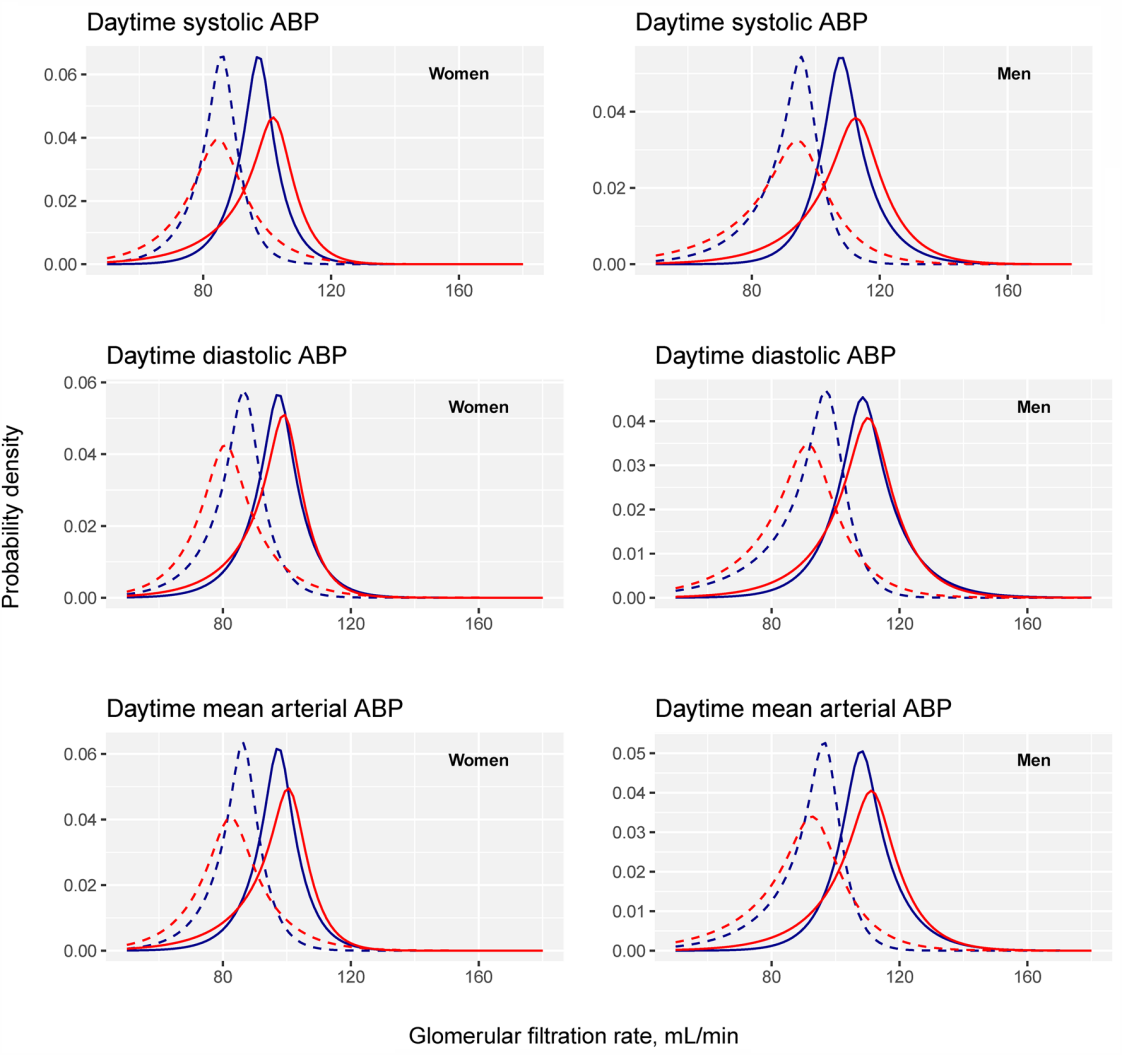
Sex-specific predicted probability density distributions of GFR at baseline (solid curves) and the longest follow-up (dashed curves) for daytime ABP components. Probability density distributions based on GAMLSS are used to show the association of ABP with the complete GFR distributions in addition to association with the mean, as in conventional regression. Separate curves are shown for the 5th (blue) and 95th (red) percentiles of the corresponding ABP component (110 and 152 mmHg for daytime SBP, 68 and 96 mmHg for daytime DBP and 83 and 114 mmHg for daytime MAP). GFR is indicated on the x-axis, and the probability density is indicated on the y-axis.

In these best fitting models, the daytime but not the nighttime ABP components, were associated with nonlinear time changes in the GFR distribution (p < 0.05) (Table 3). Accordingly, only daytime ABP was a risk factor for the time change of GFR. The predicted time changes of the four SHASH parameters at the mean of the adjustment variables for daytime ABP are plotted in Fig. S2, and the complete GFR probability density functions at baseline and the maximum follow-up of 13 years are shown in Fig. 2.

**Table 3:** P-values for the non-linear associations between ABP components and parameters of the SHASH distribution in the best fitting GAMLSS models for GFR and ABP-data in the RENIS cohort.

ABP component	SHASH parameter	P-value for association	
		Time-independent level	Time-dependent change
Daytime ABP			
Systolic	Location	0.03	0.01
	Scale	< 0.001	0.31
	Skewness	0.001	0.14
	Tailweight	Constant	Constant
Diastolic	Location	0.76	0.01
	Scale	0.02	0.40
	Skewness	0.003	0.04
	Tailweight	Constant	Constant
Mean arterial pressure	Location	0.01	0.01
	Scale	< 0.001	0.28
	Skewness	0.05	0.07
	Tailweight	Constant	Constant
Nighttime ABP			
Systolic	Location	0.06	0.31
	Scale	< 0.001	0.11
	Skewness	0.01	0.74
	Tailweight	Constant	Constant
Diastolic	Location	0.001	0.27
	Scale	< 0.001	0.07
	Skewness	Constant	Constant
	Tailweight	Constant	Constant
Mean arterial pressure	Location	0.004	0.26
	Scale	< 0.001	0.05
	Skewness	0.09	0.16
	Tailweight	Constant	Constant

Each row in the table represents the p-value for the non-linear association between the corresponding ABP component and SHASH parameter in Model G of Table S5 for all ABP components except nighttime DBP, where Model C had the best fit.

*RENIS* the Renal Iohexol-clearance Survey, *ABP* ambulatory blood pressure, *SHASH distribution* sinh-arcsinh distribution, *GAMLSS* generalized additive models for location, scale and shape, *RENIS* the Renal Iohexol-clearance Survey, *GFR* glomerular filtration rate.

**Figure 3:**
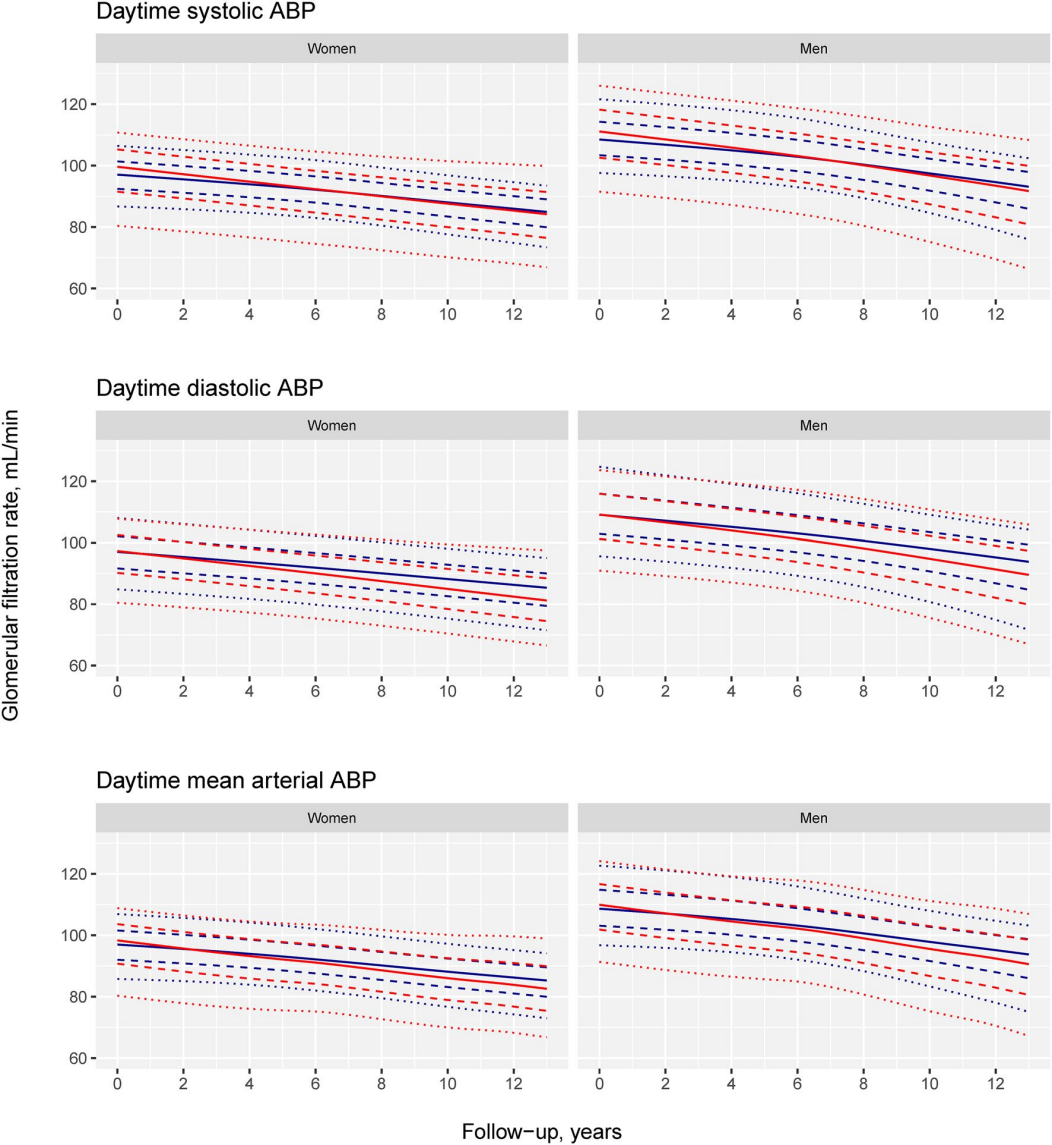
Sex-specific distributions of predicted GFR as functions of follow-up. For each plot, separate curves for the 5th (blue) and 95th (red) percentiles of the corresponding ABP component are shown (110 and 152 mmHg for daytime SBP, 68 and 96 mmHg for daytime DBP and 83 and 114 mmHg for daytime MAP). Plots for the ABP components with a statistically significant association with the time change of the GFR distribution and time are shown. The dotted lines represent the 10th and 90th percentiles, the dashed lines represent the 25th and 75th percentiles, and the solid line represents the 50th percentile of the GFR distribution. The predictions are based on the best fitting GAMLSS model in Table 3 with adjustment variables set at their baseline means and random effects set at zero.

In contrast, all of the ABP components demonstrated cross-sectional time-independent associations with the location and scale parameters (p < 0.05) (Table 3). The daytime and nighttime SBP were also associated with the skewness parameter (p < 0.05). This indicates that high ABP was associated with a wider GFR distribution, which was skewed toward a lower GFR for systolic ABP at baseline (Figs. 2, 3 and Fig. S2).

**Figure 4:**
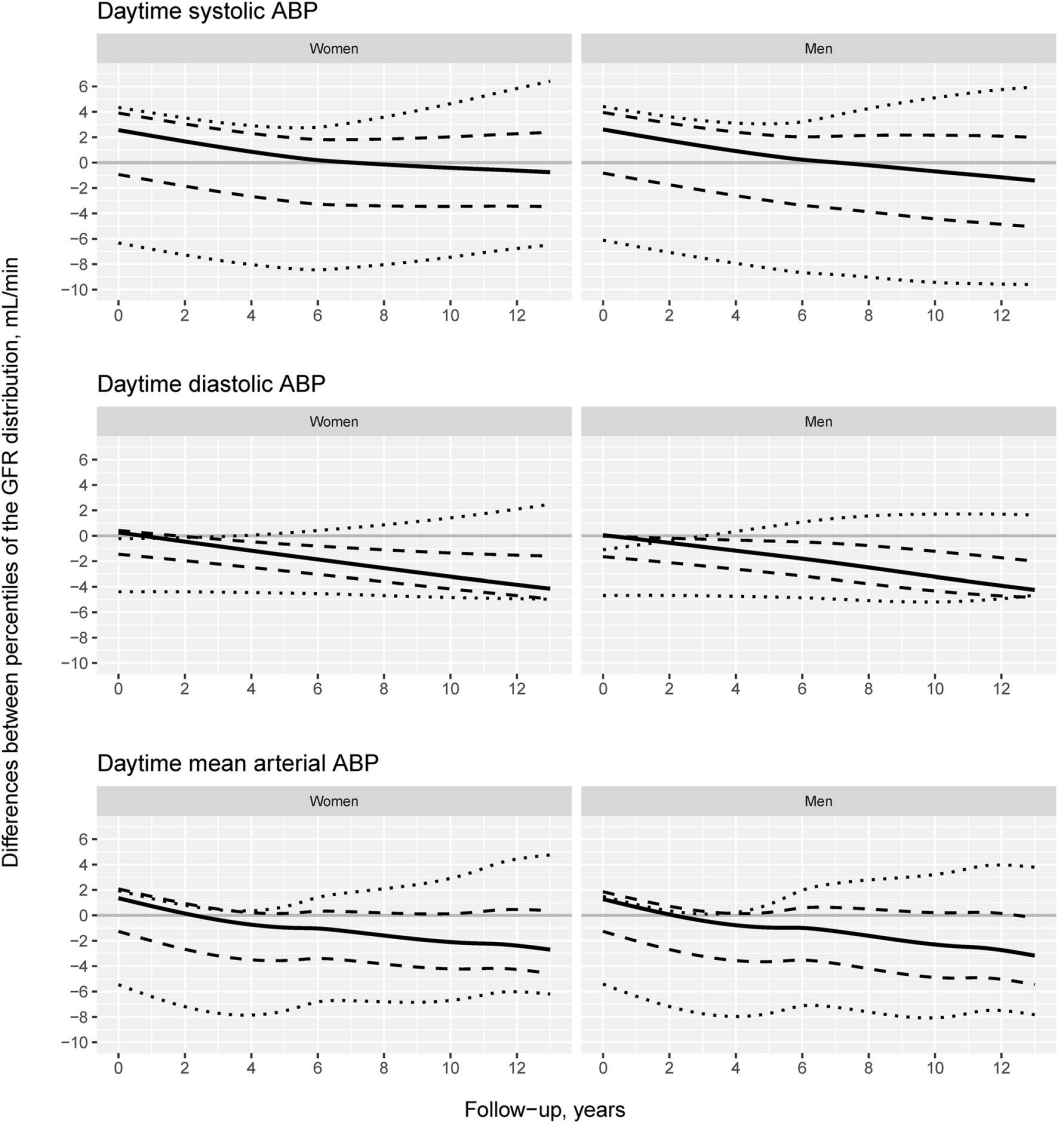
Sex-specific differences between the percentiles of GFR presented in Fig. 3 for the 95th and 5th percentiles of ABP components as functions of time. The dotted lines represent differences between the two ABP levels for the 10th and 90th percentiles, the dashed lines for the 25th and 75th percentiles and the solid line for the 50th percentile of the GFR distribution. E.g., the solid line (50th percentile of GFR) for systolic ABP in men is positive at baseline and declines with follow-up to negative values corresponding to the red solid curve (high systolic ABP) declining faster and crossing the blue solid line (low systolic ABP) in Fig. 3.

The corresponding predicted time change of the 10th, 25th, 50th, 75th and 90th percentiles of the GFR distribution for daytime ABP at the mean of the adjustment variables are shown in Fig. 3. The figure includes separate curves for the 5th and 95th percentiles of the ABP components (110 and 152 mmHg for daytime SBP, 68 and 96 mmHg for daytime DBP and 83 and 114 mmHg for daytime MAP). The difference between the GFR percentiles for these two ABP levels vs. time is plotted in Fig. 4.

At baseline, Fig. 4 demonstrates that the central part of the GFR distribution between the 25th and 75th percentiles is greater for high than for low daytime SBP. Over the follow-up period, the differences decreased for high vs. low daytime SBP, DBP and MAP by approximately 2 to 5 mL/min. This indicates a modestly steeper GFR decline for most persons with high daytime ABP. The largest difference between the ABP levels was found for the 10th percentile of the GFR distribution, but this effect was fairly constant over time, except for an increasingly negative difference in daytime SBP in men (Fig. 4). This means that high ABP confers a higher probability of GFR lower than the 10th percentile but that this risk increases over time only for daytime SBP in men. The increasingly positive difference between the 90th percentiles of the GFR distribution for all three daytime ABP components indicates that some people with high daytime ABP developed higher GFR than people with low ABP (Figs. 3 and 4).

Sensitivity analyses with office BP components in the best fitting GAMLSS in Table 3 found no statistically significant associations with the time change of the SHASH parameters (Supplementary Results, Table S5). Sensitivity analyses after exclusion of persons with antihypertensive treatment demonstrated the same pattern of statistically significant time-dependent associations with change in the daytime ABP SHASH parameters as in the total cohort (Supplementary Results, Table S6, Fig. S3).

### Ten-year probability of incident GFR less than 60 mL/min/1.73 m^2^

**Figure 5:**
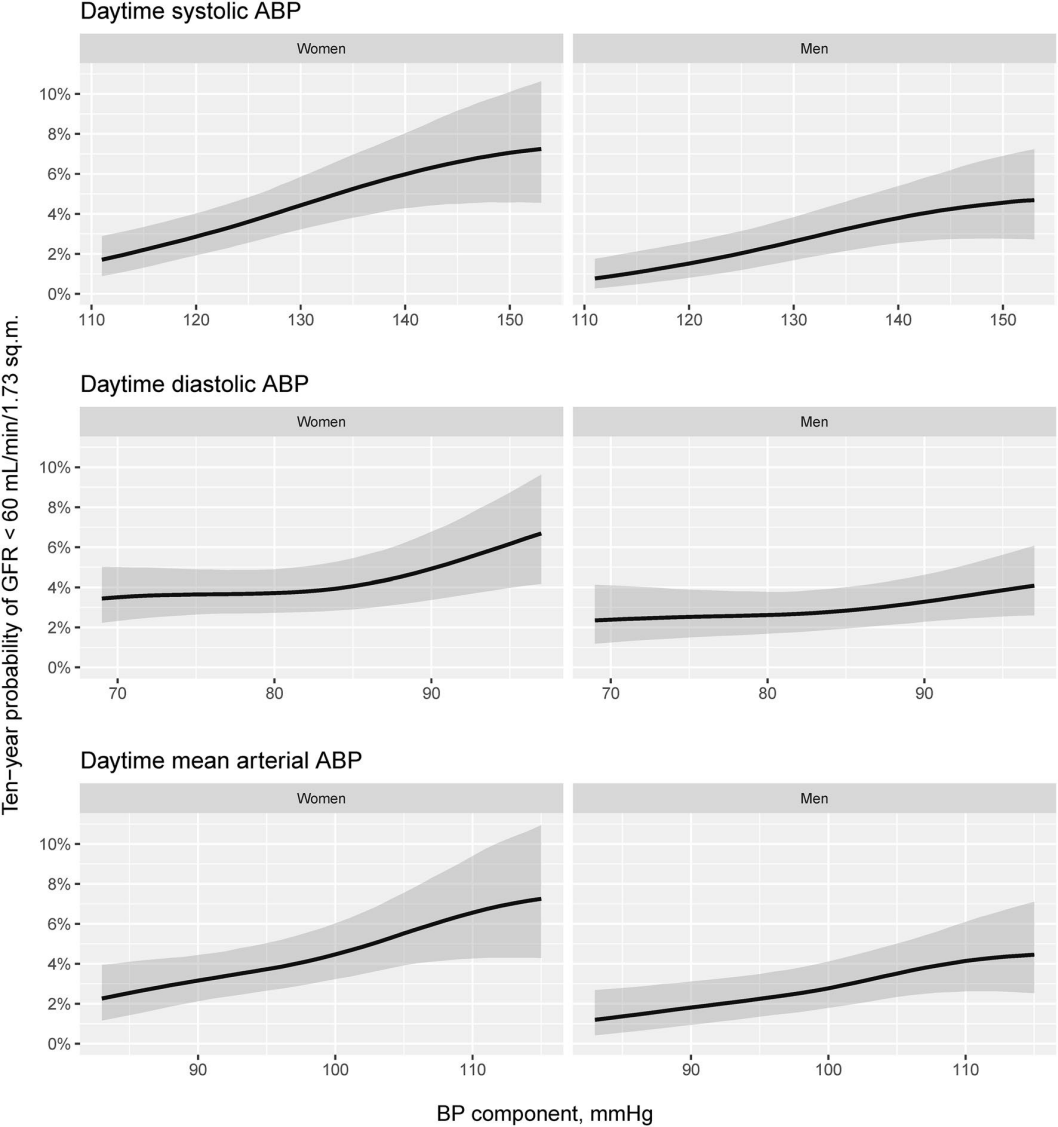
Sex-specific predicted ten-year probabilities of a GFR less than 60 mL/min/1.73 m^2^ as functions of daytime SBP, DBP and MAP. The predictions were based on the best fitting GAMLSS model in Table 3 with adjustment variables set at their baseline means and random effects set at zero. The GFR was indexed for sex-specific mean body surface area. The gray bands indicate 95% credible intervals.

Based on the best fitting GAMLSS for daytime ABP, the predicted ten-year probabilities of a GFR lower than 60 mL/min/1.73 m^2^ are shown in Fig. 5. For this purpose, the GFR used in the GAMLSS was indexed for the sex-specific mean body surface area. Simulating 5000 samples from the posterior distribution of the regression coefficients was used to construct 95% credible intervals (CI)^25^. For women, the difference in probability between the 95th and 5th percentiles for daytime SBP was 0.05 (95% CI 0.03 to 0.09), for daytime DBP 0.03 (95% CI 0.002 to 0.07) and for daytime MAP 0.05 (95% CI 0.02 to 0.09). For men, the same differences were 0.04 (95% CI 0.02 to 0.07), 0.02 (95% CI -0.01 to 0.04) and 0.03 (95% CI 0.01 to 0.06).

#### Sex

We compared the fit of GAMLSS for daytime ABP in Table 3 with and without sex-specific nonlinear functions for the interaction between ABP components and time. The AIC improved slightly only for daytime SBP (32,636 vs. 32,640), but the effects of the sex-specific terms were not statistically significant. Accordingly, we did not find evidence of sex-specific effects of ABP on the time change of the GFR distribution.

### Associations between BP and the time change of estimated GFR

Substituting eGFR_crea_, eGFR_cys_ or eGFR_cyscrea_ for GFR as the dependent variable in the fully adjusted GAMM and GAMLSS models demonstrated substantial differences between measured GFR and eGFR, and between the three different eGFRs (see Supplementary Results, Tables S7 and S8, Fig. S4).

## Discussion

This study found no association between elevated baseline BP and long-term mean GFR in the multivariable-adjusted conventional regression models (Table 2). With a distributional regression method, higher baseline daytime ABP was a risk factor for the development of a more unfavorable GFR distribution (Figs. 2, 3 and 4). We found an increased risk of a modestly accelerated decline in the central part of the GFR distribution and a small increase in the absolute risk of a low GFR between the 95th and 5th percentiles of daytime SBP (Fig. 5). Accordingly, elevated baseline daytime ABP contributed to a slightly steeper GFR decline in most people and to a small increase in the absolute risk of chronic kidney disease, defined as a low GFR.

To our knowledge, the only other longitudinal investigation of ABP and kidney function in a population-based study was McMullan et al.’s study of ABP and eGFR_crea_^32^. The authors found no association between SBP and incident CKD but did not report results for DBP. Several differences between the study population and methodology may account for the differences from our study, of which the most important were a low participation rate, the use of estimated GFR and the lack of any adjustment for antihypertensive medication^32^.

Considerable uncertainty exists about the effects of BP on GFR. Although most longitudinal observational studies have found an association between BP and subsequent GFR decline, incident CKD or ESKD^33–52^, there is no conclusive evidence from RCTs that antihypertensive treatment prevents kidney dysfunction, except in patients with CKD or diabetes^2–5^. In a meta-analysis of RCTs with 78,931 participants, BP-lowering treatment had no effect on the risk of kidney failure^6^. However, the short median follow-up of only 3.4 years of the included studies was a major limitation, which may explain why beneficial effects were difficult to detect.

We found different predicted time changes in the GFR for women and men (Figs. 3 and 5), even if there was no evidence of sex-specific BP effects on time change in the statistical models. This is a consequence of the overall nonlinear sex-specific trajectories of age-related GFR decline, which have been discussed in a previous paper^12^. Because this effect was included as an adjustment in the GAMLSS in this investigation, its predictions differed between the two sexes due to the nonlinearity of the models, although the overall pattern of changes was the same.

The time change in the GFR distribution showed a paradoxical high GFR with an increasing trend for high daytime ABP (Fig. 4). In addition to the role of high GFR or hyperfiltration as a pathogenetic factor in diabetic kidney disease^53^, there is also evidence of an association between hypertension and hyperfiltration from a recent Mendelian randomization study^54^ and from the initial drop in GFR when antihypertensive treatment is started in RCTs^55–57^. This drop has been interpreted as a beneficial effect of reducing hyperfiltration^58^. Our results suggest that hyperfiltration may persist longer than previously thought in some persons. The ultimate consequences of this are unclear.

Whereas the aim of this study was to study the time change in the GFR distribution, there were statistically significant cross-sectional time-independent associations between all of the ABP components and SHASH parameters (Table 3, Figs. 2, 3 and Fig. S2). This indicates that the associations between ABP and GFR were established at ages younger than the baseline age of our study. The association between nephron endowment and hypertension found by others suggests a congenital association but does not explain these findings^59^. The associations between ABP and GFR in younger people could have important implications for antihypertensive therapy and should be explored further.

The current study illustrates how different methods influence the results of an observational study of BP and GFR. In addition to the regression model, the method for assessing GFR is decisive: the results when using the eGFRs differ from the measured GFR and between each other (Tables S7 and S8). The explanation is probably confounders that influence both the production rate of creatinine and cystatin C and the GFR^60–65^. Accordingly, caution should be applied when using eGFR in studies of BP and GFR. Also, office BP did not identify time-dependent associations with the GFR distribution (Table S5). This suggests that ABP is a better predictor of GFR than office BP, similar to what has been found for cardiovascular outcomes. Current hypertension guidelines recognize that ABP gives important additional information for the diagnosis of hypertension both in the general population^66,67^ and in CKD patients^68^.

The most important strengths of the present study are its use of iohexol clearance and ABP, which are gold standard methods for assessing GFR and BP. To our knowledge, the duration of follow-up also exceeds all previous observational studies and RCTs studying the association between BP and GFR decline, except for two studies with a follow-up of 30 years^50,52^. Comorbidities that could mediate an indirect effect of BP on GFR may inflate the BP effect, but few previous studies excluded subjects with CVD or diabetes or adjusted for these conditions^33,35,37,38^. We studied a representative sample of the general population without CVD or diabetes, which is a further strength of our investigation.

The main limitation of this study is that inferences about causality cannot be made from observational studies. The direction of any causal connection between ABP and GFR is also uncertain, as subclinical kidney damage has been suggested as a cause of primary hypertension^69^. Although there were no linear associations between BP and GFR change in fully adjusted GAMMs, a larger study with greater statistical power may have been able to detect the small effects found with GAMLSS. ABP was only measured at baseline, and we did not consider changes in BP during follow-up. A sensitivity analysis after excluding observations with antihypertensive medications did not indicate that changes in antihypertensive medication during follow-up were important for the results. The study participants were of European ancestry, limiting the generalizability. Since we are not aware of any other population-based cohorts with ABP and serial measurements of GFR, external validation of our findings is currently not possible.

We conclude that investigations of the relationship between BP and kidney function depend critically on the methods for assessing BP and GFR as well as on the statistical methods. By using measurements of GFR and ambulatory BP in a model that relaxes the restrictive assumptions of conventional regression methods, we found that elevated daytime ABP was associated with a shift in the GFR distribution toward lower GFR. This will only be associated with a modest acceleration of GFR decline in most people but increases the risk of CKD. The genetic and environmental causes of low GFR in a minority of persons with high ABP are clinically important and should be the subject of further research. The potential for preserving GFR in these persons through antihypertensive treatment should also be explored, ideally in a long-term RCT with change in measured GFR as the endpoint.

### Supplementary Information

Supplementary Information.

## Data Availability

The data underlying this article cannot be shared publicly because this was not included in the research permission, due to ethical considerations and the privacy of individuals that participated in the study. The data can be shared on request from the corresponding author as part of research collaboration.
