# Author Correction: Ambulatory blood pressure as risk factor for long-term kidney function decline in the general population: a distributional regression approach

**DOI:** 10.1038/s41598-024-63297-0

**Published:** 2024-06-03

**Authors:** Bjørn O. Eriksen, Matteo Fasiolo, Ulla D. Mathisen, Trond G. Jenssen, Vidar T. N. Stefansson, Toralf Melsom

**Affiliations:** 1 Metabolic and Renal Research Group, UiT The Arctic University of Norway, Tromsø, Norway; 2 Section of Nephrology, Clinic of Internal Medicine, University Hospital of North Norway, Tromsø, Norway; 3 School of Mathematics, University of Bristol, Bristol, UK; 4 Department of Transplant Medicine, Oslo University Hospital and University of Oslo, Oslo, Norway

Correction to: *Scientific Reports* 10.1038/s41598-023-41181-7, published online 31 August 2023

The original version of this Article contained errors in the calculation of the time from iohexol injection to sampling for some of the measurements in the third wave of the survey, which resulted in errors in the reported iohexol-clearance measurements. As a result, Tables 2 and 3 and the Supplementary Table S6 were incorrect.

The correct and incorrect values appear below.

Incorrect Table 2.

**Table:** 

Blood pressure component	Model 1					Model 2					Model 3				
	Beta coefficient, mL/min/year (95% CI)				P-value	Beta coefficient, mL/min/year (95% CI)				P-value	Beta coefficient, mL/min/year (95% CI)				P-value
Ambulatory blood pressure, per 10 mmHg increase															
24 h															
Systolic	− 0.07	− 0.13	to	− 0.01	0.03	− 0.05	− 0.11	to	0.01	0.10	− 0.05	− 0.11	to	0.02	0.14
Diastolic	− 0.07	− 0.16	to	0.02	0.13	− 0.06	− 0.15	to	0.03	0.21	− 0.05	− 0.15	to	0.04	0.28
Mean arterial pressure	− 0.08	− 0.16	to	0.00	0.06	− 0.06	− 0.15	to	0.02	0.13	− 0.06	− 0.14	to	0.03	0.18
Daytime															
Systolic	− 0.04	− 0.10	to	0.01	0.12	− 0.03	− 0.09	to	0.03	0.29	− 0.03	− 0.08	to	0.03	0.37
Diastolic	− 0.03	− 0.12	to	0.05	0.43	− 0.03	− 0.11	to	0.06	0.56	− 0.02	− 0.11	to	0.07	0.66
Mean arterial pressure	− 0.05	− 0.12	to	0.03	0.23	− 0.03	− 0.11	to	0.04	0.40	− 0.03	− 0.11	to	0.05	0.50
Nighttime															
Systolic	− 0.07	− 0.13	to	− 0.01	0.02	− 0.05	− 0.11	to	0.01	0.08	− 0.05	− 0.11	to	0.01	0.12
Diastolic	− 0.08	− 0.17	to	0.00	0.06	− 0.08	− 0.16	to	0.01	0.09	− 0.07	− 0.16	to	0.02	0.12
Mean arterial pressure	− 0.09	− 0.17	to	− 0.01	0.03	− 0.07	− 0.15	to	0.01	0.07	− 0.07	− 0.15	to	0.01	0.10
Daytime to nighttime blood pressure dip^a^, per 0.10 increase															
Systolic	− 0.07	− 0.18	to	0.04	0.22	− 0.06	− 0.17	to	0.05	0.30	− 0.06	− 0.17	to	0.05	0.32
Diastolic	− 0.08	− 0.17	to	0.02	0.12	− 0.07	− 0.16	to	0.03	0.17	− 0.06	− 0.16	to	0.03	0.19
Office blood pressure, per 10 mmHg increase															
Systolic	− 0.05	− 0.09	to	− 0.01	0.03	− 0.04	− 0.08	to	0.00	0.07	− 0.04	− 0.08	to	0.01	0.10
Diastolic	− 0.06	− 0.14	to	0.02	0.13	− 0.06	− 0.14	to	0.02	0.15	− 0.05	− 0.14	to	0.03	0.19
Mean arterial pressure	− 0.06	− 0.13	to	0.00	0.05	− 0.06	− 0.13	to	0.01	0.08	− 0.05	− 0.12	to	0.01	0.11

Correct Table 2.

**Table:** 

Blood pressure component	Model 1					Model 2					Model 3				
	Beta coefficient, mL/min/year (95% CI)				P-value	Beta coefficient, mL/min/year (95% CI)				P-value	Beta coefficient, mL/min/year (95% CI)				P-value
Ambulatory blood pressure, per 10 mmHg increase															
24 h															
Systolic	− 0.07	− 0.13	to	− 0.01	0.02	− 0.05	− 0.11	to	0.01	0.09	− 0.04	− 0.10	to	0.01	0.14
Diastolic	− 0.05	− 0.14	to	0.03	0.23	− 0.04	− 0.13	to	0.05	0.39	− 0.03	− 0.12	to	0.06	0.49
Mean arterial pressure	− 0.07	− 0.15	to	0.01	0.08	− 0.05	− 0.13	to	0.03	0.19	− 0.05	− 0.13	to	0.04	0.27
Daytime															
Systolic	− 0.05	− 0.10	to	0.01	0.10	− 0.03	− 0.08	to	0.02	0.27	− 0.03	− 0.08	to	0.03	0.36
Diastolic	− 0.02	− 0.10	to	0.06	0.64	− 0.01	− 0.09	to	0.08	0.86	0.00	− 0.09	to	0.08	0.99
Mean arterial pressure	− 0.04	− 0.11	to	0.03	0.30	− 0.02	− 0.10	to	0.05	0.53	− 0.02	− 0.09	to	0.06	0.67
Nighttime															
Systolic	− 0.07	− 0.13	to	− 0.02	0.01	− 0.06	− 0.11	to	0.00	0.05	− 0.05	− 0.11	to	0.01	0.09
Diastolic	− 0.08	− 0.16	to	0.01	0.07	− 0.07	− 0.15	to	0.01	0.10	− 0.06	− 0.15	to	0.02	0.15
Mean arterial pressure	− 0.09	− 0.16	to	− 0.01	0.02	− 0.07	− 0.15	to	0.00	0.07	− 0.06	− 0.14	to	0.01	0.10
Daytime to nighttime blood pressure dip^a^, per 0.10 increase															
Systolic	− 0.08	− 0.19	to	0.03	0.15	− 0.07	− 0.17	to	0.04	0.21	− 0.06	− 0.17	to	0.04	0.23
Diastolic	− 0.09	− 0.18	to	0.01	0.07	− 0.08	− 0.17	to	0.01	0.10	− 0.07	− 0.17	to	0.02	0.11
Office blood pressure, per 10 mmHg increase															
Systolic	− 0.04	− 0.08	to	0.00	0.03	− 0.04	− 0.08	to	0.01	0.010	− 0.03	− 0.07	to	0.01	0.14
Diastolic	− 0.05	− 0.12	to	0.03	0.23	− 0.04	− 0.12	to	0.03	0.26	− 0.04	− 0.12	to	0.04	0.32
Mean arterial pressure	− 0.06	− 0.12	to	0.01	0.08	− 0.05	− 0.11	to	0.02	0.14	− 0.04	− 0.11	to	0.02	0.19

Incorrect Table 3.

**Table:** 

ABP component	SHASH parameter	P-value for association	
		Time-independent level	Time-dependent change
Daytime ABP			
Systolic	Location	0.01	0.02
	Scale	< 0.001	0.28
	Skewness	0.002	0.13
	Tailweight	Constant	Constant
Diastolic	Location	< 0.001	0.004
	Scale	0.01	0.21
	Skewness	0.59	0.02
	Tailweight	Constant	Constant
Mean arterial pressure	Location	0.006	0.006
	Scale	< 0.001	0.17
	Skewness	0.06	0.13
	Tailweight	Constant	Constant
Nighttime ABP			
Systolic	Location	0.04	0.49
	Scale	< 0.001	0.22
	Skewness	0.01	0.68
	Tailweight	Constant	Constant
Diastolic	Location	0.001	0.17
	Scale	< 0.001	0.08
	Skewness	Constant	Constant
	Tailweight	Constant	Constant
Mean arterial pressure	Location	0.004	0.23
	Scale	< 0.001	0.07
	Skewness	0.09	0.27
	Tailweight	Constant	Constant

Correct Table 3.

**Table:** 

ABP component	SHASH parameter	P-value for association	
		Time-independent level	Time-dependent change
Daytime ABP			
Systolic	Location	0.03	0.01
	Scale	< 0.001	0.31
	Skewness	0.001	0.14
	Tailweight	Constant	Constant
Diastolic	Location	0.76	0.01
	Scale	0.02	0.40
	Skewness	0.003	0.04
	Tailweight	Constant	Constant
Mean arterial pressure	Location	0.01	0.01
	Scale	< 0.001	0.28
	Skewness	0.05	0.07
	Tailweight	Constant	Constant
Nighttime ABP			
Systolic	Location	0.06	0.31
	Scale	< 0.001	0.11
	Skewness	0.01	0.74
	Tailweight	Constant	Constant
Diastolic	Location	0.001	0.27
	Scale	< 0.001	0.07
	Skewness	Constant	Constant
	Tailweight	Constant	Constant
Mean arterial pressure	Location	0.004	0.26
	Scale	< 0.001	0.05
	Skewness	0.09	0.16
	Tailweight	Constant	Constant

The original incorrect Supplementary Information file appears below.

The original Article has been corrected.

### Supplementary Information

Supplementary Information.

