# Supplementary material for: Author Correction: Ambulatory blood pressure as risk factor for long-term kidney function decline in the general population: a distributional regression approach
Source: Sci Rep. 2024 Jun 3;14:12712. doi: 10.1038/s41598-024-63297-0 (PMC11148025; doi:10.1038/s41598-024-63297-0)
Supplement: Supplementary file 1 — Supplementary Information. [file 41598_2024_63297_MOESM1_ESM.docx]

#

Supplementary Information

**AMBULATORY BLOOD PRESSURE AS RISK FACTOR FOR LONG-TERM KIDNEY FUNCTION DECLINE IN THE GENERAL POPULATION: A DISTRIBUTIONAL REGRESSION APPROACH**

Bjørn O. Eriksen^1,2^ M.D. Ph.D., Matteo Fasiolo Ph.D.^3^, Ulla D. Mathisen^1,2^ M.D. Ph.D., Trond G. Jenssen^1,4^ M.D. Ph.D., Vidar T.N. Stefansson^1,2^ M.D. Ph.D., Toralf Melsom^1,2^ M.D. Ph.D.

^1^Metabolic and Renal Research Group, UiT The Arctic University of Norway, Tromsø, Norway; ^2^Section of Nephrology, Clinic of Internal Medicine, University Hospital of North Norway, Tromsø, Norway; ^3^School of Mathematics, University of Bristol, United Kingdom; ^4^Department of Transplant Medicine, Oslo University Hospital and University of Oslo, Oslo; Norway

Table of Contents

[Detailed Methods 3](#_Toc120354804)

[The sinh-arcsinh (SHASH) distribution 3](#_Toc120354805)

[Supplemental Figures and Figure Legends 4](#_Toc120354806)

[Figures 6](#_Toc120354807)

[Figure S1. Examples of the SHASH probability density function. 6](#_Toc120354808)

[Figure S2. Predictions of time change of the location, scale and skewness SHASH parameters for daytime ABP 7](#_Toc120354809)

[Figure S3. Time change of differences between percentiles of GFR for persons without antihypertensive treatment 8](#_Toc120354810)

[Figure S4. Time change of difference between percentiles of eGFRcys 9](#_Toc120354811)

[Supplemental Tables and supporting information 10](#_Toc120354812)

[Sensitivity analyses 10](#_Toc120354813)

[Associations of blood pressure components with the mean GFR change rate 10](#_Toc120354814)

[Nonlinear associations of ambulatory blood pressure with the time change in the GFR distribution 10](#_Toc120354815)

[Associations between blood pressure and the time change of estimated GFR 11](#_Toc120354816)

[Associations of blood pressure components with the mean estimated GFR change rate 11](#_Toc120354817)

[Nonlinear associations of ambulatory blood pressure with the time change in the estimated GFR distribution 11](#_Toc120354818)

[Tables 12](#_Toc120354819)

[Table S1. Missing baseline study variables in RENIS. 12](#_Toc120354820)

[Table S2. Linear associations between the mean GFR change rates and BP components in GAMM after exclusion of CVD and antihypertensive medication. 13](#_Toc120354821)

[Table S3. Linear associations between the mean GFR change rates and BP components in GAMM with BSA-adjusted GFR. 14](#_Toc120354822)

[Table S4. Comparison of AIC for GAMLSS fitted to GFR and ABP data. 15](#_Toc120354823)

[Table S5. P-values for the association of office BP with GFR in GAMLSS. 16](#_Toc120354824)

[Table S6. P-values for the association of daytime ABP with GFR after exclusion of persons with antihypertensive treatment. 16](#_Toc120354825)

[Table S7. Linear associations between the mean GFR change rates and BP in GAMM with eGFR from creatinine, cystatin C or both. 18](#_Toc120354826)

[Table S8. P-values for the association of ABP with eGFR in GAMLSS. 19](#_Toc120354827)

[Supplemental References 20](#_Toc120354828)

# Detailed Methods

## The sinh-arcsinh (SHASH) distribution

In most regression methods, the error term is assumed to be distributed normally with a constant variance and a mean estimated as a function of the independent variables. For some purposes, these assumptions are too restrictive because the independent variables have effects on other aspects of the error distribution. In these cases, a more flexible alternative is the sinh-arcsinh (SHASH) distribution which has four separate parameters for location, scale, skewness and tailweight (the thickness of the tails of the distribution relative to its central part).^1^ The location and scale parameters are analogous to the mean and variance of the normal distribution, and the normal distribution is a special case of the SHASH distribution when skewness is 0 and tailweight is 1. Figure S1 shows the SHASH distribution when each of the four parameters varies relative to a standard normal distribution with mean 0 and standard deviation 1.

In general additive models for location, scale and shape (GAMLSS), the error term is assumed to follow a SHASH distribution, and each of its four parameters can be modelled as a nonlinear function of the independent variables, including observation time in longitudinal studies.^2-4^ From this, quantiles of the dependent variable as functions of time can be estimated.

# Supplemental Figures and Figure Legends

Figure S1. Examples of the sinh-arcsinh (SHASH) distribution for different values of the parameters for location (mu), scale (sigma), skewness (nu) and tailweight (tau) in separate panels. The standard normal distribution corresponding to the values mu=0, sigma=1, nu=0 and tau=1 is shown in solid black in all panels for comparison. Each panel shows the SHASH probability density function for one low (blue dashed curve) and one high (red dashed curve) value of each of the four parameters. Extreme values have been chosen to illustrate the effects. The parameter value is indicated in the corresponding color next to each curve.

Figure S2. The predicted time changes of the location, scale and skewness SHASH parameters at the mean of the adjustment variables for daytime ABP for the best fitting GAMLSS. The 5^th^ (blue) and 95^th^ (red) percentiles of the ABP components are shown. The tailweight parameter was constant in these models.

Figure S3. Sex specific differences between percentiles of GFR for the 95^th^ and 5^th^ percentile of daytime ABP components as functions of observation time for persons who did not receive any antihypertensive treatment during the study period (n=958). The predictions are based on the GAMLSS model in Table 3 estimated for this subgroup. The adjustment variables were set at their baseline means and the random effects at zero. The dotted lines represent differences between the two BP levels for the 10^th^ and 90^th^ percentile, the dashed lines for the 25^th^ and 75^th^ percentile and the solid line for the 50^th^ percentile of the GFR distribution.

Figure S4. Sex-specific differences between the percentiles of eGFR_cys_ for the 95th and 5th percentile of ABP components as functions of time. The dotted lines represent differences between the two ABP levels for the 10th and 90th percentile, the dashed lines for the 25th and 75th percentile and the solid line for the 50th percentile of the GFR distribution.

# Figures

## Figure S1. Examples of the SHASH probability density function.


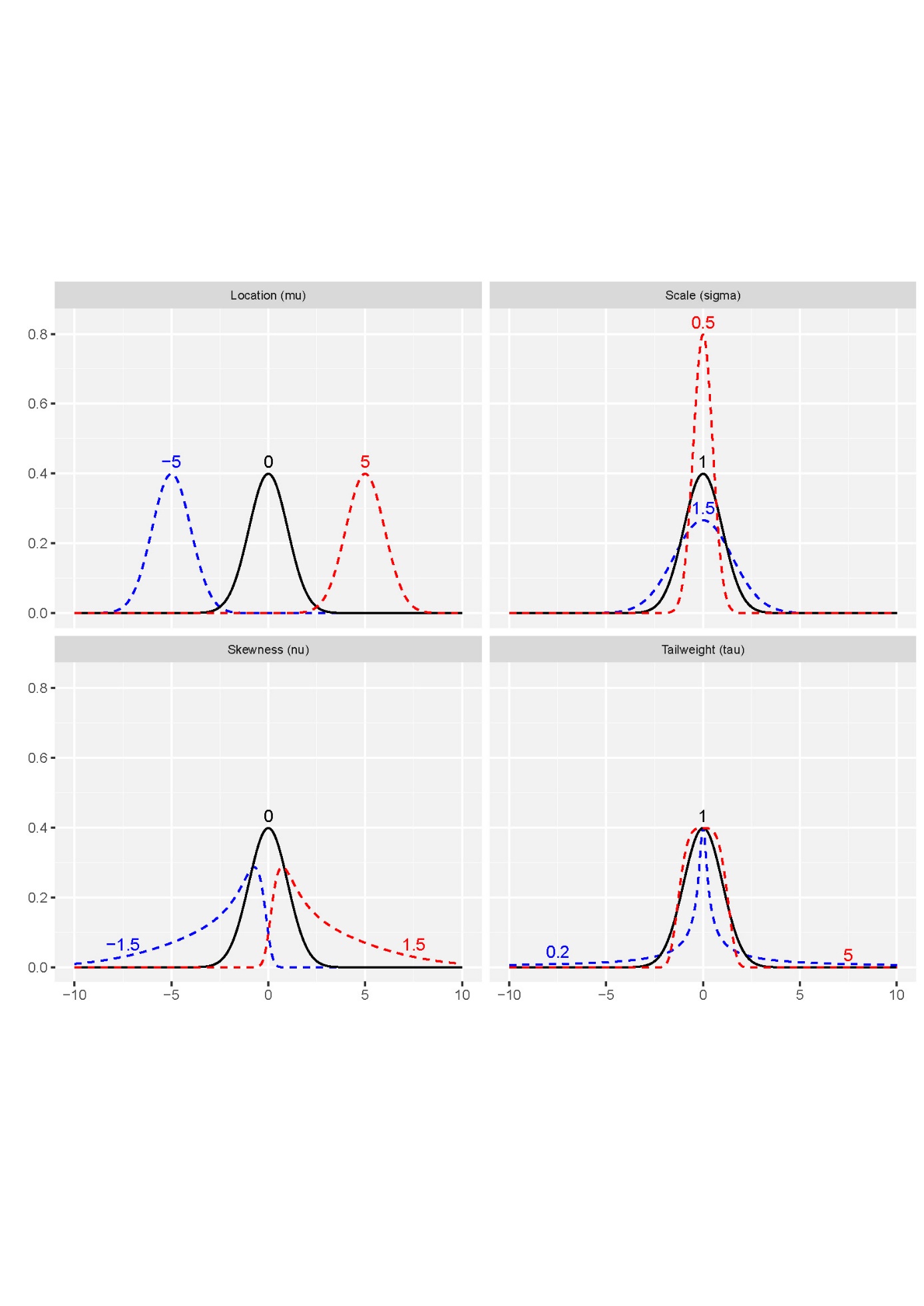


## Figure S2. Predictions of time change of the location, scale and skewness SHASH parameters for daytime ABP

## Figure S3. Time change of differences between percentiles of GFR for persons without antihypertensive treatment

## Figure S4. Time change of difference between percentiles of eGFRcys

# Supplemental Tables and supporting information

## Sensitivity analyses

### Associations of blood pressure components with the mean GFR change rate

Sensitivity analyses with the fully adjusted GAMMs in Model 2, Table 2 were performed after excluding 62 observations with self-reported CVD at any of the follow-up examinations and after excluding 631 persons with antihypertensive treatment at baseline or follow-up with essentially the same results (Table S2). Substituting body surface area adjusted GFR for absolute GFR as the dependent variable and removing body weight and height from the independent variables in the fully adjusted models also gave similar results (Table S3).

### Nonlinear associations of ambulatory blood pressure with the time change in the GFR distribution

When office SBP, DBP and MAP were substituted for daytime ABP components in the best fitting GAMLSS in Table 3, there were similar cross-sectional associations with the time-independent parameters for location and scale as for daytime ABP, but there were no statistically significant associations with the time change of the SHASH parameters (Table S5). This indicates that office BP was not associated with the time change of the GFR distribution in the study population.

When persons with antihypertensive treatment at baseline or follow-up were excluded, the GAMLSS demonstrated the same pattern of statistically significant time-dependent associations with change in the daytime ABP SHASH parameters as in the total cohort, but with an additional association with the time change of the skewness parameter for daytime MAP (p=0.01) (Table S6). The overall decreasing trends of the percentile difference curves were similar to the total cohort for the 25^th^, 50^th^ and 75^th^ percentiles of the GFR distribution but they were more variable for the 10^th^ and 90^th^ percentiles (Figure S3).

## Associations between blood pressure and the time change of estimated GFR

### Associations of blood pressure components with the mean estimated GFR change rate

Substituting eGFR_crea_, eGFR_cys_ or eGFR_cyscrea_ for absolute GFR as the dependent variable and removing body weight and height from the independent variables in the fully adjusted GAMM (Model 2, Table 2) demonstrated substantial differences between the association between ABP, eGFR_crea_ and eGFR_cyscrea_ vs. between ABP and eGFR_cys_ (Table S7). Whereas the results for eGFR_crea_ and eGFR_cyscrea_ were very similar to those for the measured GFR, eGFR_cys_ had statistically significant and lower mean GFR change rates for most of the BP components.

### Nonlinear associations of ambulatory blood pressure with the time change in the estimated GFR distribution

When eGFR_crea_, eGFR_cys_ or eGFR_cyscrea_ were substituted for absolute GFR as the dependent variable and body weight and height were removed from the independent variables in the best fitting GAMLSS in Table 3, there were no associations between any of the daytime ABP components and the time change of the eGFR_crea_ distribution (p>0.05). The location, scale and skewness parameters of the distributions of both eGFR_cys_ and eGFR_cyscrea_ were all associated with all of the daytime ABP components (p<0.05), except for the scale parameters for daytime SBP and daytime MAP for eGFR_cys_ (Table S8). The time change of differences between percentiles for high and low ABP is shown for eGFR_cys_ in Figure S4. In contrast to the measured GFR (Figure 4), the percentile differences are almost exclusively negative and of lesser magnitude. The differences for eGFR_cyscrea_ were similar.

# Tables

## Table S1. Missing baseline study variables in RENIS.

## Table S2. Linear associations between the mean GFR change rates and BP components in GAMM after exclusion of CVD and antihypertensive medication.

##

## Table S3. Linear associations between the mean GFR change rates and BP components in GAMM with BSA-adjusted GFR.

## Table S4. Comparison of AIC for GAMLSS fitted to GFR and ABP data.

## Table S5. P-values for the association of office BP with GFR in GAMLSS.

## Table S6. P-values for the association of daytime ABP with GFR after exclusion of persons with antihypertensive treatment.

## Table S7. Linear associations between the mean GFR change rates and BP in GAMM with eGFR from creatinine, cystatin C or both.

## Table S8. P-values for the association of ABP with eGFR in GAMLSS.

# Supplemental References

1 Jones, M. C. & Pewsey, A. Sinh-arcsinh distributions. *Biometrika* **96**, 761-780 (2009).

2 Rigby, R. A. & Stasinopoulos, D. M. Generalized Additive Models for Location, Scale and Shape. *J R Stat Soc Ser C Appl Statist* **54**, 507-554 (2005).

3 Wood, S. N. *Generalized Additive Models. An Introduction with R.* Second edn, (CRC Press, 2017).

4 Fasiolo, M., Nedellec, R., Goude, Y. & Wood, S. N. Scalable visualisation methods for modern Generalized Additive Models. *J Comput Graph Stat* **29**, 78-86 (2020).
